# Identification of *O*-GlcNAcylated proteins in *Plasmodium falciparum*

**DOI:** 10.1186/s12936-017-2131-2

**Published:** 2017-11-29

**Authors:** Mattis Kupferschmid, Moyira Osny Aquino-Gil, Hosam Shams-Eldin, Jörg Schmidt, Nao Yamakawa, Frédéric Krzewinski, Ralph T. Schwarz, Tony Lefebvre

**Affiliations:** 1 Institute for Virology, Laboratory of Parasitology, Philipps-University, Marburg, Germany; 2 Univ. Lille, CNRS, UMR 8576, UGSF, Unité de Glycobiologie Structurale et Fonctionnelle, 59000 Lille, France; 3 Instituto Tecnológico de Oaxaca, Tecnológico Nacional de México, Oaxaca, Mexico; 4 Centro de Investigación UNAM-UABJO, Universidad Autónoma Benito Juárez de Oaxaca, Oaxaca, Mexico

**Keywords:** *Plasmodium falciparum*, *O*-GlcNAcylation, Proteomics, α-Tubulin, Hsp70, Glycolysis

## Abstract

**Background:**

Post-translational modifications (PTMs) constitute a huge group of chemical modifications increasing the complexity of the proteomes of living beings. PTMs have been discussed as potential anti-malarial drug targets due to their involvement in many cell processes. *O*-GlcNAcylation is a widespread PTM found in different organisms including *Plasmodium falciparum*. The aim of this study was to identify *O*-GlcNAcylated proteins of *P. falciparum*, to learn more about the modification process and to understand its eventual functions in the Apicomplexans.

**Methods:**

The *P. falciparum* strain 3D7 was amplified in erythrocytes and purified. The proteome was checked for *O*-GlcNAcylation using different methods. The level of UDP-GlcNAc, the donor of the sugar moiety for *O*-GlcNAcylation processes, was measured using high-pH anion exchange chromatography. *O*-GlcNAcylated proteins were enriched and purified utilizing either click chemistry labelling or adsorption on succinyl-wheat germ agglutinin beads. Proteins were then identified by mass-spectrometry (nano-LC MS/MS).

**Results:**

While low when compared to MRC5 control cells, *P. falciparum* disposes of its own pool of UDP-GlcNAc. By using proteomics methods, 13 *O*-GlcNAcylated proteins were unambiguously identified (11 by click-chemistry and 6 by sWGA-beads enrichment; 4 being identified by the 2 approaches) in late trophozoites. These proteins are all part of pathways, functions and structures important for the parasite survival. By probing clicked-proteins with specific antibodies, Hsp70 and α-tubulin were identified as *P. falciparum O*-GlcNAc-bearing proteins.

**Conclusions:**

This study is the first report on the identity of *P. falciparum O*-GlcNAcylated proteins. While the parasite *O*-GlcNAcome seems close to those of other species, the structural differences exhibited by the proteomes provides a glimpse of innovative therapeutic paths to fight malaria. Blocking biosynthesis of UDP-GlcNAc in the parasites is another promising option to reduce *Plasmodium* life cycle.

## Background

The estimated malaria death rate declined by over 50% during the last 15 years. This has been the result of a considerable international effort to globally provide potent anti-malarial drugs, insecticide-impregnated bed nets and indoor residual spraying. However, malaria still caused over 400,000 deaths in 2015 worldwide, most of them being young children. History taught that if alternatives in malaria treatment are lacking the tide of success in eradication may turn quickly. The 1955 WHO-Global Eradication of Malaria Programme was declared to have failed its aims after 14 years. The emergence of resistances against chloroquine and DDT, considered at this time as the two pillars in the fight against malaria, led to the end of the progress and even in some regions to a severe relapse [1, 2].

Indeed the emergence of resistance against artemisinins which are part of the current first-line treatment [3], and of insecticide-resistant Anopheles mosquitoes [4], is threatening the current progress. Hence it remains important to push forward the search for alternative targets of anti-malarial therapies. Therefore, more information about basic regulatory functions of the parasite’s life cycle is needed. Post translational modifications (PTMs) form a huge group of regulators that are not encoded by DNA and for some that control cell functions. A group of PTMs expressed by *Plasmodium* including phosphorylation and acetylation but that neglected glycosylation has been described and discussed as potential drug targets [5]. *O*-GlcNAcylation that was discovered by Hart’s team in the early 80s [6], is a PTM involved in many different cell processes. The authors undertook the radiolabelling of GlcNAc residues exhibited by surface glycoproteins and were amazed when they found that in fact a majority of the amino sugar moieties occur inside the cell [7]. This was in contrast to the dogma claiming that glycosylation only occurs on cell membranes or inside organelles. The authors further found that this uncommon form of glycosylation, unlike to the complex branched sugar-trees of *N*-glycosylation, consisted of one single GlcNAc residue directly linked through a β-linkage on the hydroxyl group of serine or threonine [5]. In ensuing experiences it was shown that contrary to other known forms of glycosylation, *O*-GlcNAcylation is a dynamic modification [8]. It cycles on and off proteins thanks to a pair of enzymes: the *O*-GlcNAc transferase (OGT) adds the monosaccharide [9] and the *O*-GlcNAcase (OGA) catalyzes its hydrolysis [10]. Given these features differences *O*-GlcNAcylation endows some functions that are distinct from complex glycosylations such as transcriptional processes, protein synthesis or proteasomal degradation. *O*-GlcNAcylation is akin to phosphorylation with which it can compete at the same or at neighbouring amino acid residues [11]. Deregulations in *O*-GlcNAcylation processes are found in different diseases including metabolic disorders [12], as well as neoplastic [13] and neurodegenerative diseases [14].

OGTs are expressed in mammals [9], insects [15], fishes [16] and worms [17] as well as in plants [18], bacteria [19], viruses [20] and protists [21]. The latter has been a rather neglected field with few more than a handful of publications considering *Toxoplasma gondii*, *Cryptosporidium parvum* and *Plasmodium falciparum* [21–23]. In *P. falciparum*, *O*-GlcNAcylation was first reported by Dieckman-Schuppert et al. [22] in 1993 by using the same approach than Torres and Hart [7]. More recently, Perez-Cervera et al. visualized *P. falciparum O*-GlcNAc-modified proteins by western blot [23]. In the present study, the level of UDP-GlcNAc, the donor of the GlcNAc moiety, was measured and, albeit low, confirmed that *P. falciparum* possesses its own nucleotide sugar stock. Different tools were used to confirm the occurrence of *O*-GlcNAcylation in *P. falciparum*, and *O*-GlcNAcylated proteins were purified and enriched which enabled to identify 13 of them by mass spectrometry.

## Methods

### *Plasmodium falciparum* culture and stage separation

The 3D7 strain was obtained from Behring Co. (Marburg, Germany) and was cultured according to Trager and Jensen [24] in human A+ erythrocytes and in a RPMI medium containing neomycin, glutamine, Na_2_CO_3_ and human fresh frozen plasma (FFP) to reach a final haematocrit of 5% (v/v). The culture bottles were incubated in an environment containing 5% (v/v) O_2_, 5% (v/v) CO_2_ and 90% (v/v) N_2_ at 37 °C. The medium was changed every 1–2 days. The culture was microscopically monitored by Giemsa-stained smears. Contamination with Mycoplasma was ruled out by PCR control. The erythrocytes were separated by using SuperMACS columns (Miltenyi Biotec GmbH, Bergisch Gladbach, Germany) in a magnetic field, hereby erythrocytes infected with late trophozoites were isolated from uninfected red blood cells and erythrocytes containing parasites in the ring form in a magnetic field [25]. The parasites were extracted by lysis of the erythrocyte’s membranes using a saponin buffer [26].

### Protein extraction

The parasites were lysed on ice in the following buffer: 10 mM Tris/HCl, 150 mM NaCl, 1 mM EDTA, 1% (v/v) Triton X-100, 0.5% (w/v) sodium deoxycholate, 0.1% (w/v) SDS, pH 7.4. After vigorous mixing and sonication, the lysate was centrifuged at 20,000*g* for 10 min at 4 °C. The pellet was discarded and the supernatant saved for further analyses. For incubation with PNGase F parasites were lysed in the following buffer: 10 mM Tris/HCl, 1 mM EDTA, 1 mM EGTA and 0.5% (v/v) Triton X-100, pH 7.5. For sWGA (succinylated-wheat germ agglutinin)-beads enrichment, a hypotonic buffer was used; the composition was as follows: 10 mM Tris/HCl, 1 mM MgCl_2_, 10 mM NaCl, 15 mM 2-mercaptoethanol, pH 7.2. A cocktail of proteases inhibitors was added to each buffer.

### SDS-PAGE and western blot

Proteins were resolved on 8 or 10% SDS-PAGE and either brilliant blue stained or electroblotted onto nitrocellulose sheet. Equal loading and transfer efficiency were checked using Ponceau red staining. Membranes were saturated in 5% (w/v) non-fatty milk in Tris buffered Saline (TBS)-Tween [15 mM Tris, 140 mM NaCl, 0.05% (v/v) Tween, pH 8.0] for 1 h or in 5% (w/v) bovine serum albumin (BSA) in TBS-Tween overnight. The primary antibodies (anti-*O*-GlcNAc, anti-tubulin and anti-HSP70) were incubated overnight at 4 °C. Then nitrocellulose membranes were washed three times for 10 min each in TBS-Tween and incubated with horseradish peroxidase-labelled secondary antibodies for 1 h or with Streptavidin-HRP over 45 min. Finally, three washes of 10 min each were performed with TBS-Tween and the detection was carried out with enhanced chemiluminescence (GE Healthcare).

### Succinylated-WGA protein enrichment

Prior to sWGA-beads enrichment, 100 µg of proteins were incubated with PNGase F (New England Bio-Labs) according to the manufacturer’s instructions. Then, proteins were incubated with sWGA beads overnight in a buffer containing 10 mM Tris/HCl, 100 mM NaCl, 0.4% (w/v) sodium deoxycholate, 0.3% (w/v) SDS and 0.2% (w/v) Nonidet P-40, pH 7.5. Beads were washed four times using the same buffer without and then with free GlcNAc. After boiling in Laemmli buffer proteins were run by SDS-PAGE.

### Labelling of *O*-GlcNAcylated proteins by click-chemistry

**Fig. 1:**
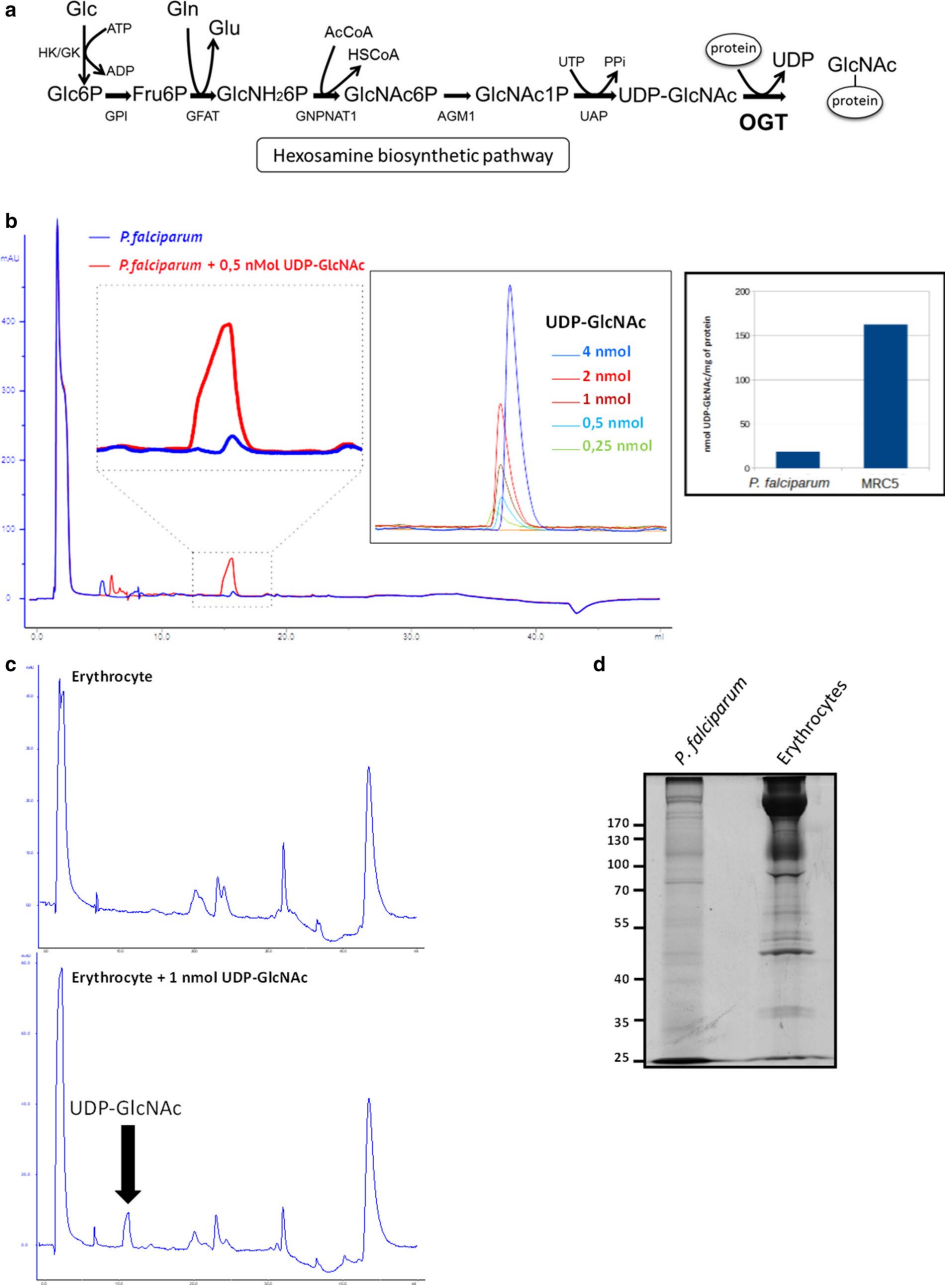
Monitoring of the UDP-GlcNAc content in *Plasmodium falciparum.* UDP-GlcNAc is the end-product of the hexosamine biosynthetic pathway (**a**). Among other GlcNAc transferases, UDP-GlcNAc is the substrate of OGT (adapted from Aquino-Gil et al. [58]). HK/GK, hexokinase/glucokinase; GPI, glucose-6-phosphate isomerase; GFAT, glutamine: fructose-6-phosphate amidotransferase; GNPNAT1, GlcNH_2_-6-phosphate *N*-acetyltransferase 1; AGM1, phospho-GlcNAc mutase 1; UAP, UDP-GlcNAc pyrophosphorylase; OGT, *O*-GlcNAc transferase. Late trophozoite UDP-GlcNAc contents were analysed by using a FPLC system (AKTA purifier; ProPAC-PA1 column; detection carried out at 256 nm) as described in the materials session (blue line) (**b**). A co-injection of the sample with 0.5 nmol UDP-GlcNAc is shown in red. Increasing amounts of standard UDP-GlcNAc were injected for calibration (top insert). The quantity of UDP-GlcNAc was normalized for 1 mg of proteins for comparison with MRC5 cells (bottom insert). **c** Uninfected erythrocytes were analysed alone according to their UDP-GlcNAc content or co-injected with 1 nmol of UDP-GlcNAc. **d** Total proteins from *P. falciparum* and erythrocytes were run using SDSPAGE (right panel). The gel was stained with brilliant blue

Labelling of *O*-GlcNAc-bearing proteins by GalNAz and biotin alkyne were done using the Click-it *O*-GlcNAc enzymatic labelling system and the Click-it glycoprotein detection kit (biotin alkyne) according to the manufacturer’s instructions (Fischer Scientific) (Fig. 1) [27]. Bovine α-crystallin was used as a positive control. After labelling, proteins were precipitated using the methanol/chloroform kit protocol and resuspended in 50 µL of Tris/HCl pH 8.0 containing 0.1% (m/v) SDS. 700 µL of enrichment buffer [1% (v/v) Triton X-100 and 0.1% (w/v) SDS in PBS] was added to the sample before incubating with 50 µL of avidin-coupled beads (1 h, 4 °C). Avidin-bound proteins were collected, washed three times with the enrichment buffer, resuspended in Laemmli buffer and boiled. Controls of labelling and enrichment followed the same procedure except that the chemical labelling with UDP-GalNAz was omitted.

### Mass spectrometry

Specific bands were excised from brilliant blue stained gels. The pieces of gel were reduced using a 50:50 dilution of 50 mM ammonium bicarbonate (Bic) (HPLC Grade, Prolabo) and acetonitrile (ACN) (Sigma A) followed by 100% (v/v) ACN. They were then reduced in 20 mM DTT (Sigma) in 50 mM Bic and alkylated in 100 mM iodoacetamide (Bio-Rad) in 50 mM Bic. After washing in ACN/Bic the bands were digested with 100–200 ng trypsin gold (Promega) in 25 mM Bic. Extraction was done using 45% (v/v) ACN and 10% (v/v) formic acid (Sigma). The extracted peptides were purified using C_18_-Zip-Tip cones using 0.1% (v/v) formic acid for washing and 50:50 ACN/0.1% (v/v) formic acid to elute the purified sample. The nano-LC MS/MS analysis was performed on a HPLC system with two LC-20AD nano-flow LC pumps, a SIL-20 AC auto-sampler and a LC-20AB micro-flow LC pump (Shimadzu, Kyoto, Japan) connected to an ion-trap mass spectrometer (amaZon ETD, Bruker Daltonics, Bremen, Germany) equipped with a Captive Spray ion source. Hystar (Version 3.2, Bruker Daltonics, Bremen, Germany) was used to couple and control Shimadzu CBM-20A module (Shimadzu, Kyoto, Japan) for MS acquisition for all experiments.

Trapping and desalting of the peptides was carried out on nano trapping column (Zorbax 300SB-C18, 5 µm, 0.3 × 5 mm, Agilent) using 0.05% (v/v) trifluoroacetic acid solution for 10 min at a flow rate of 10 µL/min. After back-flushing from the trapping column, peptides were separated on a reversed-phase column, ACQUITY UPLC^®^ M-Class Peptide BEH C18 Column (1.7 μm, 130 Å, 100 × 0.75 mm i.d., Waters) using an acetonitrile/0.1% (v/v) formic acid gradient from 15 to 50% (v/v) acetonitrile within 30 min. Fine tuning was achieved using the smart parameter setting (SPS) option for m/z 800, compound stability and trap drive level were set at 100%. Optimization of the captive spray source resulted in dry gas temperature, 150 °C, dry gas, 3.0 L/min, capillary voltage, − 1200 V, end plate offset, 0 V. The MS1 and MS2 ion detection window was set at m/z 100–1700. The five highest nonsingly charged peaks in each MS1 spectrum were automatically fragmented through collision-induced dissociation (CID).

### Data processing

The LC–MS/MS data was analysed using DataAnalysis 4.1 software (Bruker Daltonics). Within this program, the function Compounds-AutoMS(n) was used to generate 1200 compound spectra, and these data were exported as a mascot generic file. Protein identification through primary sequence database searching was performed using the MASCOT search algorithm (MASCOT free version; Matrix Science, London, UK). The following MASCOT settings were used: taxonomy: *P. falciparum*; database: SwissProt; enzyme: trypsin; fixed modifications: carbamidomethyl (C); variable modifications: oxidation (M, HW), deamidation (NQ), phosphorylation (ST), pyro-Glu (N-term E), and HexNAc (ST); max missed cleavages: 1; MS1 peptide tolerance: 0.6 Da; MS/MS tolerance: 0.6 Da.

### UDP-GlcNAc pools monitoring

UDP-GlcNAc pools measurement using FPLC system. The procedure was described by Guinez et al. [28]. Briefly, parasites, erythrocytes or MRC5 cells were lysed in 0.5 mL of hypotonic buffer. 25 μL of 1 M HCl was added to the lysate for proteins acidification. The solution was passed through a column containing 1.5 mL of Dowex 50WX2-400 in its H+ form. Washes were performed with 3 mL of 18 MΩ water. The unbound fraction and washes were collected on ice and adjusted with 250 μL of 1 M Tris/HCl to pH 8.0. 1.5 mL of the diluted fraction was injected using a ProPAC-PA1 column (4 × 250 mm) on AKTA Purifier 10 system. The elution programme analysis was the following: 20 mM Tris/HCl, pH 9.2 (solution A) for 1 min; elution gradient for 29 min with 85% of A and 15% NaCl at 2 M (solution B); plateau of 5 min in these conditions; an elution gradient of 10 min until 100% of B was reached; a plateau at 100% of B was maintained for 5 min. The column was then re-equilibrated in 100% of A. The flow rate was of 0.8 mL/min. Detection was performed at 256 nm.

## Results

### Determination of the UDP-GlcNAc level displays by *Plasmodium falciparum*

In order to learn about the resources the parasite can draw on for its *O*-GlcNAcylation processes, the level of UDP-GlcNAc was assayed (Fig. 1). This nucleotide sugar is the substrate of OGT and, therefore, it is crucial for *O*-GlcNAcylation processes. UDP-GlcNAc is the end product of the hexosamine biosynthesis pathway (Fig. 1a), a metabolic pathway highly sensitive to nutrient levels and glucose sensitizing in somatic cells. Besides *O*-GlcNAcylation it is used for the biosynthesis of complex glycans and glycosylphosphatidyl-inositol (GPI)-anchors. To determine the UDP-GlcNAc pool, late trophozoites lysates were processed as described under the methods session (Fig. 1b). The calibration was done by measuring the response of increasing amounts of standard UDP-GlcNAc by high performance anion exchange chromatography (HPAEC). The procedure was then applied to late trophozoites. The same samples were assayed for their protein contents and ratio of nmol of UDP-GlcNAc per milligram of proteins calculated. Although the UDP-GlcNAc level is around tenfold lower when compared to human fibroblasts (MRC5 cell line was used as a control), the study showed that there is a pool of precursor for the *O*-GlcNAcylation processes managed by *P. falciparum*. To discard the possible contamination of *P. falciparum* with erythrocytes components two controls were performed; UDP-GlcNAc was monitored in uninfected erythrocytes and protein profiles from the Apicomplexan and erythrocytes were compared. No trace of UDP-GlcNAc was detected in the erythrocyte that is devoid of most of its organelles and lost the capacity to synthesis proteins and to perform complex glycosylation (Fig. 1c). This observation is also in accordance with [29] that does not mention the existence of UDP-GlcNAc in this cell. Lastly, SDS-PAGE revealed that the two cell types display distinct protein profiles proving that no cross-contamination occured during the preparation of *P. falciparum* (Fig. 1d).

### Visualization of the *Plasmodium falciparum O*-GlcNAcome

**Fig. 2:**
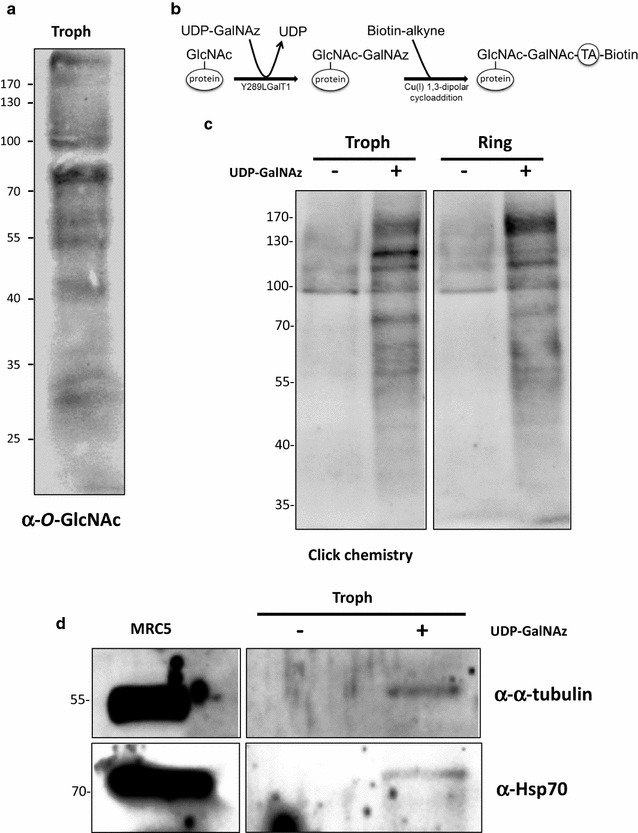
A large panel of proteins expressed by *P. falciparum* are *O*-GlcNAcylated. *P. falciparum* proteins were analysed by western blot using the anti-*O*-GlcNAc antibody RL2 (**a**) or after labelling of the *O*-GlcNAc-bearing proteins by GalNAz and biotin alkyne using the Click-it™ *O*-GlcNAc enzymatic labelling system and the Click-it™ Glycoprotein detection kit as described in the “Methods” section (**b**). After labelling *P. falciparum O*-GlcNAcylated proteins were separated by SDS-PAGE and western blot analyses were performed using HRP-labelled avidin to test the efficiency of the labelling (**c**). After enzymatic and chemical labelling, *P. falciparum O*-GlcNAcylated proteins were enriched on avidin-beads, separated by SDS-PAGE, electroblotted onto nitrocellulose membrane and analysed by western blot according to their α-tubulin and Hsp70 contents. The clicked-proteins were enriched on avidin-beads and analysed by Western blot by according to their alpha-tubulin and Hsp70 antibodies (**d**)

To visualize the occurrence of the *O*-GlcNAc modification in *P. falciparum* approaches based on enzymatic labelling (Click-chemistry) and antibodies were used. Trophozoites *O*-GlcNAcylated proteins were exposed to the anti-*O*-GlcNAc antibody RL2 (Fig. 2a). This antibody was first described to recognize eight nuclear pore complex proteins and was found to actually identify *O*-GlcNAc moieties of those proteins [30]. Afterwards, crude extracts of late trophozoites and parasites in their ring stage were processed as described under the "Methods" section. A mutant galactosyltransferase was used to add azidomodified galactose onto *O*-GlcNAc residues. In a second step biotin-alkyne was added to bind to the azido group into a cycloaddition reaction. This conjugate makes the *O*-GlcNAcylated proteins detectable with avidin-labelled peroxidase (Fig. 2b). This technique is very sensitive, however a negative control (UDP-GalNAz is omitted) is needed to distinguish natural biotinylation from biotin-labeling. Clicked-proteins were further run by SDS-PAGE, transferred onto nitrocellulose and stained with peroxidase-labelled avidin (Fig. 2c). The trophozoites and the ring stage parasites display some differences in the protein profiles. In analogy to previous findings [23] the parasites proteome is vastly *O*-GlcNAcylated.

Clicked-proteins were enriched on avidin-beads and analysed by western blot by using anti-α-tubulin and anti-Hsp70 antibodies (Fig. 2d) since both proteins are heavily expressed in many living systems and that their *O*-GlcNAcylation is well-recognized in varying systems [23, 31–33]. Accordingly, this approach revealed that α-tubulin and Hsp70 are also *O*-GlcNAc-modified in *P. falciparum*.

This approach attempting to identify *P. falciparum O*-GlcNAcylated proteins is limited since it implies to predict which protein may be modified and to have available antibodies that react with *P. falciparum* proteins. Then, the approaches that were selected combine enrichment of glycosylated proteins and mass spectrometry identification of the candidates.

### Identification of *O*-GlcNAcylated proteins in *Plasmodium falciparum*

**Fig. 3:**
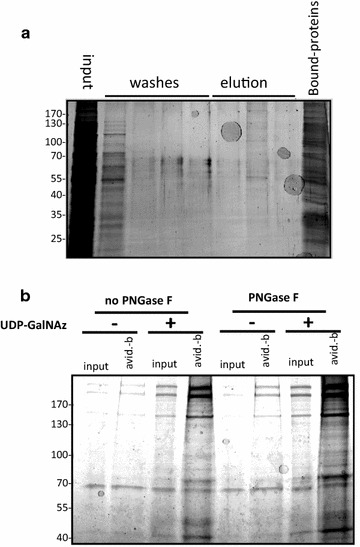
Enrichment of *P. falciparum O*-GlcNAcylated proteins was  performed by utilizing sWGA in conjunction with PNGase F digestion (**a**). As a second approach, after labelling of *P. falciparum O*-GlcNAcylated proteins by Click-chemistry as described previously, clicked proteins were treated or not with PNGase F, purified and enriched using avidin beads for further identification by mass spectrometry (**b**). Gels were brilliant blue-stained. Bands were excised, submitted to trypsin digestion for further identification

Lectins are proteins that bind sugar moieties. In plants, they act as a defense against insects and other predators [34]. Wheat germ agglutinin has a high specificity towards GlcNAc and sialic acid [35]. But, when the lectin is succinylated (sWGA) it loses its affinity towards sialic acid, making it a highly specific detector of terminal GlcNAc [36]. Besides *O*-GlcNAcylation, *N*-linked glycosylated, oligosaccharides on the cell surface, often contain GlcNAc partly in terminal positions. To avoid those false positives protein extracts were previously treated with PNGase F an enzyme that cleaves the oligosaccharides at their link to asparagine in the proteins [37]. The sWGA-labelled beads allowed to purify proteins that were resolved by SDS-PAGE. Proteins were stained with brilliant blue (Fig. 3a). In a last approach, the Click-iT™ system was used to biotin-label *O*-GlcNAcylated proteins after treatment with PNGase F (Fig. 3b). Besides the detection by western blot with avidin-labelled HRP, proteins were enriched using avidin-labelled agarose beads, the eluate was loaded on a gel and brilliant blue stained (Fig. 3b). Bands of the proteins enriched using either sWGA or Click-iT™ were excised from the gels. Proteins were digested with trypsin and analysed by tandem mass spectrometry. Protein identification was performed using the Mascot search engine against the SwissProt database restricted to *P. falciparum* taxonomy. This allowed the identification of 13 proteins (Table 1). The identified proteins can be classified according to their function. They take part in glycolysis, in the organization of cytoskeleton, act as chaperones, or have other cellular functions. The Click-iT™ method showed to be more sensitive compared to the sWGA-method, with 11 in contrast to 6 identified proteins. Note that Hsp70 first detected by probing Clicked-proteins with specific antibodies was also found with the Click-iT™ method. Alpha-tubulin—and beta-tubulin—was also detected (by Click-chemistry and WGA-enrichment) but with a low percentage of sequence recovery.

**Table 1:** *Plasmodium falciparum O*-GlcNAcylated proteins identified by mass spectrometry

UniProtKB (accession number)	Score	Coverage	Mass	Matches	Sequences	Full name	Confirmed by using sWGA	[Ref.] (biological model)
Click-chemistry								
ACT1_PLAF7	169	0.12	42,072	8 (7)	4 (4)	Actin-1	X	[27] (*Xenopus laevis*); [59] (human breast cancer cell line MCF7)
ACT2_PLAF7	30	0.07	42,977	4 (4)	3 (3)	Actin-2		[59] (human breast cancer cell line MCF7)
ALF_PLAFA	1566	0.36	40,479	39 (38)	15 (14)	Fructose-bisphosphate aldolase		[60] (*Rattus norvegicus*)
CH60_PLAFG	96	0.08	79,738	6 (3)	5 (3)	Chaperonin CPN60, mitochondrial		[61] (rat pancreatic b-cell line Rin-m5f)
EF1A_PLAFK	732	0.23	49,238	25 (21)	10 (9)	Elongation factor 1-alpha	X	[27] (*Xenopus laevis*); [59] (human breast cancer cell line MCF7)
ENO_PLAFA	694	0.18	49,015	20 (16)	8 (5)	Enolase		[27] (*Xenopus laevis*); [59] (human breast cancer cell line MCF7); [60, 62] (*Rattus norvegicus*)
GRP78_PLAFO	1864	0.27	72,845	49 (46)	22 (21)	78 kDa glucose-regulated protein homolog	X	ER-specific *O*-GlcNAc (eOGT, EGF repeat-specific *O*-GlcNAc-transferase)?
HSP70_PLAFA	1889	0.31	74,754	66 (59)	21 (20)	Heat shock 70 kDa protein = 2	X	[59] (human breast cancer cell line MCF7); [49] (human hepatocarcinoma derived cell line HepG2)
HXK_PLAFA	38	0.07	56,166	4 (1)	3 (1)	Hexokinase		No previous report
OAT_PLAFD	519	0.19	46,938	15 (10)	9 (6)	Ornithine aminotransferase		No previous report
PGK_PLAF7	562	0.29	45,569	21 (21)	11 (11)	Phosphoglycerate kinase		[57] (*Rattus norvegicus*)
sWGA								
MYOA_PLAFB	224	0.08	93,017	10 (5)	7 (2)	Myosin-A		[54, 60] (*Rattus norvegicus*)
KC1_PLAF4	69	0.09	38,036	5 (3)	3 (1)	Casein kinase I		No previous report

## Discussion

The *O*-GlcNAcylation in *P. falciparum*, that belongs to Apicomplexa, a phylum grouping obligate intracellular parasites, was suggested in a previous paper that described the PTM in *T. gondii* [23]. Therefore, here is the very first work reporting proteomics data regarding the *O*-GlcNAcome in *P. falciparum*. By a combination of different approaches fourteen *O*-GlcNAcylated proteins expressed by *P. falciparum* were identified. When compared to classical animal cell lines usually called upon for proteomics approaches, the difficulty to identify Apicomplexan *O*-GlcNAcylated proteins stems from the fact that it is necessary to culture high amounts of parasites (so as to produce enough quantities of proteins) and that the level of *O*-GlcNAcylation of *P. falciparum* is relatively low. The combination of these two obstacles renders the project challenging and explains why so few candidates were found in this work.

Four of the identified proteins are enzymes of the glycolysis pathway (while Hexokinase is not specific to glycolysis). Owing to the parasites lack of a complete citric acid cycle glycolysis is its only source of ATP [38]. Hsp70 and α-tubulin were identified by western blot; interestingly the former is in the list of the proteins found by mass spectrometry. Alpha-tubulin was also identified but with a low percentage of coverage; this poor rank can be ascribed to a problem of extraction of the tryptic peptides from the gel or by their masking by trouble components during the MS analysis, it is why it was not include in this list. Both Hsp70 and α-tubulin have already been described to be *O*-GlcNAcylated in different species, including *T. gondii* for Hsp70 [23], and have been discussed as potential drug targets in *P. falciparum*.

The hyper-*O*-GlcNAcylation of α-tubulin inhibits its ability to polymerize and form microtubules [31]. Due to the crucial role of tubulins as part of the cytoskeleton (intracellular transport of proteins and vesicles, mitotic spindle that pulls apart the chromosomes during division), microtubules have been largely discussed as a drug target in anti-malaria therapies. Inhibiting microtubule polymerization has been shown to lead to disruption of mitotic division of schizonts [39], decrease invasion of erythrocytes [40], interrupt development of gametozytes [41] and block liver stage [42]. The microtubules are targets of anticancer medication for decades, it is though not possible to use those drugs against *P. falciparum* due to its low susceptibility to classical microtubule-inhibitors. The concentrations needed would be equally toxic to human cells [43]. Influencing *P. falciparum* *O*-GlcNAcylation might be an alternative option to intervene in the parasite microtubule polymerization. Using the YinOYang algorithm, an online prediction server that allows the bioinformatic estimation of potential *O*-GlcNAcylation sites, for α-tubulin one site with very high *O*-GlcNAcylation score (S178) and three sites with high score (S271, S277, T361) were predicted. Ji et al. identified a specific *O*-GlcNAcylated site in the protein sequence of human α-tubulin by mass spectrometry [31]. The predicted S178 lies within this site. Sequence alignment showed that the entire protein sequence and also this specific site is highly preserved in *P. falciparum*.

Heat shock proteins are chaperone proteins, their main function is to facilitate the correct folding of proteins and assembly of oligomers. In heat shocked cells they can bind proteins, maintaining their solubility [43]. Hsp70 is involved in various other processes including protein unfolding and translocation [44], degradation of proteins [45], and proinflammatory signal transduction [46]. Its role in protein transport from the nucleus to the apicomplexan has shown to be crucial; inhibition of this traffic with 15-deoxyspergualin in *P. falciparum* cultures leads to the parasites death [47]. In that context, O-GlcNAcylation was discussed to act as a signal for nuclear residence [48], Hsp70 is *O*-GlcNAcylated in human cells [48–52], and beyond that it is able to bind *O*-GlcNAc acting as a lectin [49, 52]. Lastly the expression of Hsp70 is influenced by the cellular *O*-GlcNAcylation level [53]. Despite its importance for the parasites survival, it proves to be difficult to use as a drug target due to the proteins high conservation between human and *P. falciparum*. In the YinOYang algorithm for *P. falciparum*-Hsp70 one site with very high potential to be *O*-GlcNAcylated and/or phosphorylated at T49 and five other sites with high potential of *O*-GlcNAcylation (S92, T170, T286, S394, T443) were predicted.

Surprisingly, no nuclear pore proteins or other commonly *O*-GlcNAc-proteins (except chaperones and glycolytic enzymes) were found herein. These discrepancies can be related to the lack or to the low level of *O*-GlcNAcylation of these proteins in *P. falciparum*, and to a detection limitation. In contrast, actin was easily identified in the proteomics analyses. Actin was previously found to be *O*-GlcNAcylated [27, 54] and it is crucial for the motility of the parasite and for invasion of host cell [55]. It is not yet known whether *O*-GlcNAcylation promotes or hampers actin polymerization but, if true, *O*-GlcNAcylated-actin could be an interesting target to limit *P. falciparum* infection and dissemination.

The *O*-GlcNAcylation patterns observed in the two different stages of *P. falciparum*, trophozoite and ring, while showing similarities also harbor differences notably for proteins having an apparent molecular weight up to 100 kDa (Fig. 2b). These differences may result from a distinct expression of either OGT and/or of its protein substrates, and from the concentration of UDP-GlcNAc that can vary from one stage to another. This detection of UDP-GlcNAc—albeit relatively low—shows that the parasite possesses its own donor for *O*-GlcNAcylation processes. This observation is not completely surprising since it is known that *P. falciparum* synthesizes GPIs that need UDP-GlcNAc as the donor for the glucosamine moiety characteristic of these complex structures [56].

Recently, Maria Mota’s group proposed on rodent malaria models that, despite the lack of nutrient-sensing pathways in *P. falciparum*, the parasite can sense nutrients fluctuations of the host cell [57]. Thus, it is shown that the transcriptional programming of *P. falciparum* obeys to modifications of dietary through the parasite kinase KIN that orchestrates the response to caloric restriction. Interestingly, caloric restriction reduced parasitaemia whereas supplementation of glucose and re-feeding reversed this effect. Due to the pivotal position of the nutrient-sensor UDP-GlcNAc at the crossroad of metabolic pathways, it is tempting to propose that nutrients drive *P. falciparum* blood-stages parasites growth. Experiments in which *P. falciparum* was cultured with increasing concentrations of thiamet-G, a potent inhibitor of OGA, were done. No significant decrease in parasitaemia was observed. Nevertheless, since nothing is known neither about OGT nor OGA in *P. falciparum*, doubts remain about the efficacy of the small-molecule inhibitor in the parasite. In this way, given that OGT has not yet been detected in *P. falciparum*, neither by using immunological methods nor by data-searching with sequence alignment [23], there remains two options how *P. falciparum O*-GlcNAcylates its proteins. Either it possesses an OGT that is different in its sequence compared to other OGTs in plants and animals, or it imports the host OGT what seems unlikely in view of the membrane barriers the enzyme had to cross. The first assumption seems more likely, in consideration of the unlikeliness of an OGT being active in erythrocytes, given that they do not have any active protein biosynthesis. As discussed in the introduction session, due to the emergence of resistances against the sesquiterpenic artemisinin derivatives it is urgent to develop new therapeutics against *P. falciparum*. Blocking OGT seems compromise because of the doubts that remain regarding its occurrence, but one possible avenue would be to impinge the parasite hexosamine biosynthetic pathway (Fig. 1a). Nevertheless, many efforts have to be made in understanding the different enzymes of this metabolic pathway before envisaging the discovery of a new class of anti-malarial drugs. However, the identification of some of the parasite *O*-GlcNAc-bearing proteins provides the base for further research on the modification functions in cellular processes.

## Conclusions

Although the occurence of *O*-GlcNAcylation in *P. falciparum* was clearly demonstrated by Dieckmann-Schuppert et al. who used radioactive methods in crude preparations of the parasite [22], no individual *O*-GlcNAcylated proteins were identified. After the advent of modern mass-spectrometry coupled with bioinformatic methods a profound reinvestigation became possible. Here, isolated parasites were analysed using non-radioactive quantitative methods. The identification of individual *O*-GlcNAcylated proteins of different families was achieved; they are involved in glycolysis, protein folding, and cell shape, equivalent to their already known mammalian counterparts.

The origin of the *O*-GlcNAc transferase is not certain as no equivalent gene sequences could be found in the PlasmoDB data bank suggesting a fundamental structural difference. Thus, inhibitors might be found that inhibit interaction of *P. falciparum O*-GlcNAc transferase with its partner molecules without interfering with the human *O*-GlcNAc transferase.
